# Metabolomics analysis reveals perturbations of cerebrocortical metabolic pathways in the *Pah^enu2^* mouse model of phenylketonuria

**DOI:** 10.1111/cns.13214

**Published:** 2019-08-31

**Authors:** Li‐Hua Lu, Zheng‐Xiang Xia, Jia‐Lin Guo, Ling‐Ling Xiao, Yong‐Jun Zhang

**Affiliations:** ^1^ Department of Neonatology Shanghai First Maternity and Infant Hospital, Tongji University School of Medicine Shanghai China; ^2^ Department of Pharmacy, Shanghai Engineering Research Center of Tooth Restoration and Regeneration School & Hospital of Stomatology, Tongji University Shanghai China; ^3^ Department of Neonatology Obstetrics and Gynecology Hospital of Fudan University Shanghai China; ^4^ Department of Neonatology Xinhua Hospital, Shanghai Jiaotong University School of Medicine Shanghai China

**Keywords:** cerebral cortex, metabolic pathway, metabolomics, phenylketonuria

## Abstract

**Aims:**

Phenylketonuria (PKU), which is caused by mutations in the phenylalanine hydroxylase (*PAH*) gene, is one of the most common inherited diseases of amino acid metabolism. Phenylketonuria is characterized by an abnormal accumulation of phenylalanine and its metabolites in body fluids and brain tissues, subsequently leading to severe brain dysfunction. Various pathophysiological and molecular mechanisms underlying brain dysfunction in PKU have been described. However, the metabolic changes and their impacts on the function of cerebral cortices of patients with PKU remain largely unknown.

**Methods:**

We measured the levels of small molecule metabolites in the cerebrocortical tissues of PKU mice and wild‐type control mice using liquid chromatography‐mass spectrometry (LC‐MS)‐based metabolome analysis. Differential metabolites were further subjected to metabolic pathway and enrichment analysis.

**Results:**

Metabolome analysis revealed 35 compounds among 143 detected metabolites were significantly changed in PKU mice as compared to those in their wild‐type littermates. Metabolic pathway and enrichment analysis of these differential metabolites showed that multiple metabolic pathways, including phenylalanine, tyrosine, and tryptophan biosynthesis; valine, leucine, and isoleucine biosynthesis; alanine, aspartate, and glutamate metabolism; purine metabolism; arginine and proline metabolism and methionine metabolism, were impacted in the cerebral cortices of PKU mice.

**Conclusions:**

The data revealed that multiple metabolic pathways in cerebral cortices of PKU mice were disturbed, suggesting that the disturbances of the metabolic pathways might contribute to neurological or neurodevelopmental dysfunction in PKU, which could thus provide new insights into brain pathogenic mechanisms in PKU as well as mechanistic insights for better understanding the complexity of the metabolic mechanisms of the brain dysfunction in PKU.

## 1 INTRODUCTION

Phenylketonuria (PKU) is one of the most prevalent autosomal recessive disorders of amino acid metabolism, resulting from a severe deficiency in the activity of the catalytic enzyme phenylalanine hydroxylase (*PAH*). Phenylalanine hydroxylase dysfunction results in abnormal accumulation of phenylalanine and its metabolites in the plasma, cerebrospinal fluid, and brain tissues, such as the cerebral cortices, subsequently causing neurological and neuropsychiatric disturbances in untreated patients with PKU and even in early treated phenylketonuric patients.^1^, ^2^, ^3^ These abnormal disturbances mainly include intellectual disability, impaired emotional regulation, and neurocognitive dysfunction.^2^, ^3^ Clinical and experimental investigations have highlighted the neurotoxic role of phenylalanine at pathological concentrations in phenylketonuric patients and experimental models of PKU. Adler‐Abramovich et al^4^ observed that phenylalanine self‐assembles into amyloid‐like deposits and shows cytotoxicity in the hippocampus of PKU model mice and in parietal cortex brain tissue from patients with PKU. Li and colleagues^5^ discovered that phenylalanine suppresses neurite outgrowth and induces rat cortical neuronal death in vitro by decreasing the expression of the brain‐derived neurotrophic factor at both mRNA and protein levels. Moreover, Zhang and Schlegel^6^, ^7^ demonstrated decreased dendritic arborization of cortical neurons in cultures, reduced dendritic length, and loss of synapses in hippocampal slice cultures after phenylalanine treatment. Despite long‐term numerous clinical and experimental studies, the exact pathogenetic mechanisms underlying phenylalanine‐evoked impairment of brain function in this neurometabolic disorder remain elusive; in particular, few studies have focused on its metabolic mechanisms.

Metabolomics is a new platform of systems biology, enabling the simultaneous quantitative measurement in complex biological samples of numerous metabolites. As a powerful analytical approach, recently, metabolomics has been increasingly applied in the fields of novel disease biomarker discovery, disease action mechanisms, and evaluation of drug efficacy and toxicity.^8^ In addition, brain metabolomics has been used for understanding the pathology and identifying potential biomarkers of neurodegenerative diseases in both animal models and human postmortem tissues due to the fact that metabolic changes in the brain are more likely to reflect disease etiology than metabolic changes in peripheral biofluids.^9^ Thus, a better understanding of the metabolic pathways in brain tissues in PKU may be beneficial for understanding the pathogenesis of PKU and for revealing the potential mechanisms underlying neurological dysfunction or damage in PKU. Therefore, the aim of our study was to characterize the metabolic disturbances in the cerebrocortical tissues in a mouse model of PKU using a metabolomics approach and to elucidate their potential association with brain dysfunction or damage.

## 2 MATERIALS AND METHODS

### 2.1 Reagents and chemicals

Distilled water was prepared by the Milli‐Q purification system. Liquid chromatography‐mass spectrometry grade methanol (MeOH) and acetonitrile (ACN) were purchased from Honeywell.

### 2.2 PKU mice

Heterozygous *Pah^enu2+/−^* mice were purchased from Jackson Laboratory, bred monogamously and maintained in our specific‐pathogen‐free (SPF) animal center. The mice were genotyped by tail clipping, DNA extraction and sequencing (Figure S1). All the mice were kept on a 12‐hour light/dark cycle (lights on from 8:00‐20:00) with ad libitum access to food and water. Male *Pah^enu2−/−^* (PKU) and *Pah^enu2+/+^* (wild‐type) mice were used for this study at the age of 4 weeks. All the mice used in the experiment received humane care in compliance with institutional and national guidelines for health and care of experimental animals, and the experimental protocols were reviewed and approved by the Ethics Committee of Xinhua Hospital, Shanghai Jiaotong University School of Medicine.

### 2.3 Metabolite extraction and LC‐MS analysis

Cerebral cortices of the wild‐type and PKU mice were rapidly excised and snap‐frozen with liquid nitrogen. Twenty milligrams of mouse cortical samples were homogenized with 200 μL of H_2_O and five ceramic beads using the homogenizer. To extract all metabolites and precipitate the proteins, 800 μL MeOH/ACN (1:1, v/v) was added into the tissue lysates, vortexed for 30 seconds, sonicated for 10 minutes, and centrifuged at 14 000 *g* for 15 minutes at 4°C. Afterward, the supernatants containing metabolites were transferred to a new tube and dried in a vacuum concentrator. Finally, the extracted metabolites were reconstituted in 100 μL ACN/H_2_O (1:1, v/v) for quality control and LC‐MS analysis. All metabolite information was retrieved and processed by MRMAnalyzer as previously described.^10^

### 2.4 Principle component analysis

Principle component analysis was performed on the abundance of all 143 metabolites by using the standard R function “procomp.” The relative PCA plot was mainly generated by “ggfortify” and “cluster” package.

### 2.5 Metabolic pathway analysis

Over representation analysis (ORA) and metabolic pathway enrichment analysis were performed on 35 significantly changed metabolites from the total of 143 metabolites by using the R package “MetaboAnalystR” developed by Xia and Wishart.^11^

### 2.6 Statistical analysis

Student's *t*‐test was performed on each metabolite using SPSS software. Differences were considered significant at the value of *P* < .05. Metabolic pathway analyses were performed by using MetaboAnalystR, applying the hypergeometric algorithm for ORA and the relative‐betweenness centrality algorithm for pathway topology analysis.

## 3 RESULTS

### 3.1 Intergroup differences in metabolite profiles in mice revealed by principal component analysis

**Figure 1:**
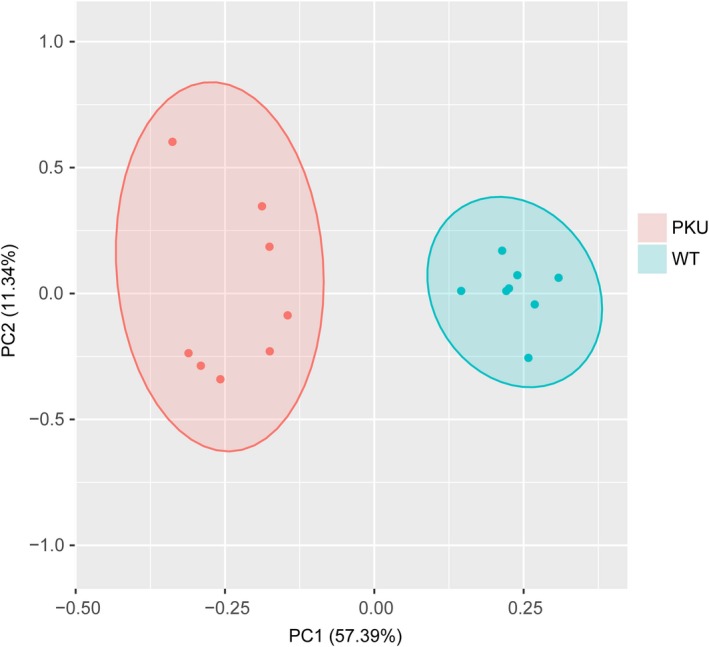
The PCA score plots of the wild‐type and PKU groups (n = 8 mice/group). Principal component analysis (PCA) score plots are displayed for each group. PKU mice are shown as orange circles, and wild‐type mice are green circles. As shown in the figure, obvious visual separation was observed between the metabolic profiles of cerebral cortex of wild‐type mice and those of PKU mice. PKU, phenylketonuria; WT, wild‐type

Principal component analysis (PCA) models are depicted as score plots and consist of two synthetic variables, PC1 and PC2, which account for the greatest proportion of the total variance and the second greatest proportion of the total variance orthogonal to PC1, respectively. The PCA score plots in Figure 1 showed that the scores belonging to the PKU mice grouped distinctly separately from the scores of the wild‐type controls in the tested cortical tissue samples. This clearly indicates that significant differences in the metabolome content exist between the wild‐type and PKU mice.

### 3.2 Identification of differential metabolites in cerebral cortices of PKU mice

**Figure 2:**
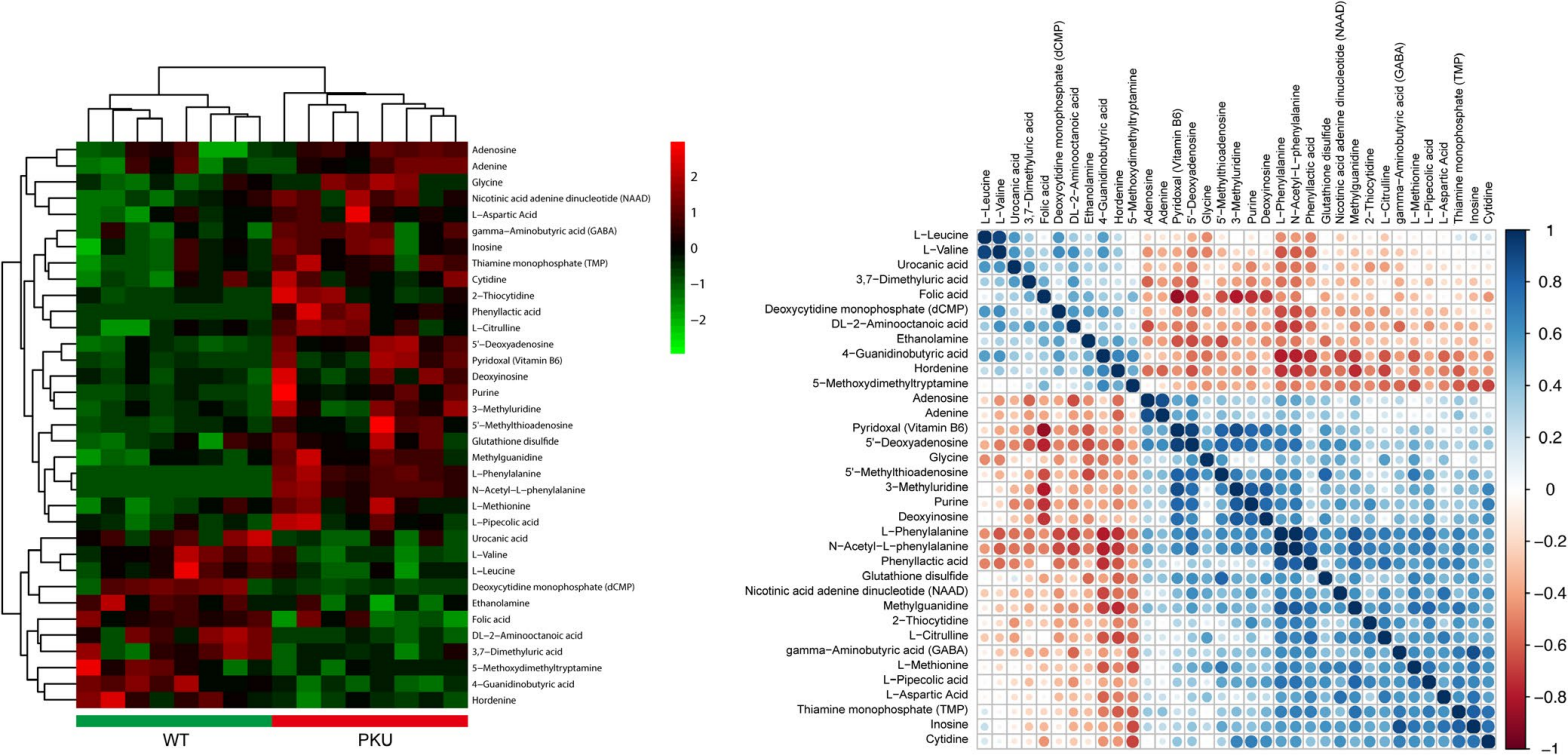
The heat map of differential metabolites in cerebral cortices of the wild‐type and PKU mice. Left, metabolic profiling of cortical samples. Rows and columns indicate samples and metabolites, respectively. The heat map shows the differential metabolite levels in the cerebral cortex of wild‐type and PKU mice (green: lowest; red: highest; black: mean). Right, the correlation matrix of differential metabolites. The color saturation of red or blue represents the negative or positive correlation coefficients between metabolites, respectively. PKU, phenylketonuria; WT, wild‐type

Metabolic profiles of cortical samples from the wild‐type control and PKU mice were acquired by using LC‐MS. Additionally, to explore the differential changes of the 143 identified metabolites between groups, we compared the concentration of each identified metabolite. Finally, a total of 35 metabolic compounds that were significantly altered in the cortical tissues of PKU mice were identified (Table 1), including l‐phenylalanine, l‐valine, l‐aspartic acid, purine, and l‐methionine. Furthermore, the differentially abundant metabolites were visualized as a heat map, which clearly showed changes in identified metabolites in the cerebral cortices between the wild‐type control and PKU groups (Figure 2). In addition, correlation analysis (Figure 2) showed a good correlation between these identified metabolites.

**Table 1:** Significantly altered metabolites in cerebral cortices of PKU mice as compared with the wild‐type mice (n = 8 mice/group)

Metabolite name	Database ID	*P*‐value^a^	FC^b^	Trend^c^
l‐Phenylalanine	HMDB00159	.0000	15.3439	↑
N‐Acetyl‐l‐phenylalanine	HMDB00512	.0000	47.6065	↑
4‐Guanidinobutyric acid	HMDB03464	.0003	0.7138	↓
5′‐Deoxyadenosine	HMDB01983	.0006	2.1202	↑
Methylguanidine	HMDB01522	.0020	1.8503	↑
Phenyllactic acid	HMDB00779	.0021	32.9758	↑
l‐Valine	HMDB00883	.0029	0.7060	↓
dl‐2‐Aminooctanoic acid	HMDB00991	.0037	0.6484	↓
Hordenine	HMDB04366	.0052	0.5861	↓
gamma‐Aminobutyric acid	HMDB00112	.0054	1.2056	↑
Deoxycytidine monophosphate	HMDB01202	.0068	0.4268	↓
l‐Citrulline	HMDB00904	.0072	1.7418	↑
Ethanolamine	HMDB00149	.0103	0.7753	↓
Nicotinic acid adenine dinucleotide	HMDB01179	.0110	1.8231	↑
Purine	HMDB01366	.0122	2.4007	↑
Pyridoxal (Vitamin B6)	HMDB01545	.0125	3.6546	↑
Thiamine monophosphate	HMDB02666	.0154	1.3278	↑
Urocanic acid	HMDB00301	.0162	0.7678	↓
5′‐Methylthioadenosine	HMDB01173	.0165	1.5250	↑
Adenosine	HMDB00050	.0176	1.5429	↑
l‐Methionine	HMDB00696	.0195	1.4764	↑
l‐Aspartic acid	HMDB00191	.0228	1.4243	↑
Inosine	HMDB00195	.0245	1.2425	↑
Folic acid	HMDB00121	.0275	0.7067	↓
2‐Thiocytidine		.0297	3.3411	↑
3‐Methyluridine	HMDB04813	.0304	1.6563	↑
Glycine	HMDB00123	.0311	2.0476	↑
Glutathione disulfide	HMDB03337	.0331	1.3049	↑
l‐Leucine	HMDB00687	.0354	0.7779	↓
Deoxyinosine	HMDB00071	.0360	1.6883	↑
3,7‐Dimethyluric acid	HMDB01982	.0365	0.7705	↓
l‐Pipecolic acid	HMDB00716	.0367	1.6126	↑
Adenine	HMDB00034	.0479	1.3524	↑
Cytidine	HMDB00089	.0489	1.3039	↑
5‐Methoxydimethyltryptamine	HMDB02004	.0490	0.4675	↓

Abbreviation: PKU, phenylketonuria.

a *p*‐Value was calculated using the Student's *t*‐test. The metabolites were given in the order of decreasing statistical significance.

b FC, fold change. Values > 1.0 indicate that levels were higher in the cortical tissues of PKU mice; values < 1.0 indicate that levels were lower in the cortical tissues of PKU mice.

c “↑” indicates that the levels were higher in the cortical tissues of PKU mice; “↓” indicates that the levels were lower in the cortical tissues of PKU mice.

### 3.3 Metabolic pathway analysis of differential metabolites

**Figure 3:**
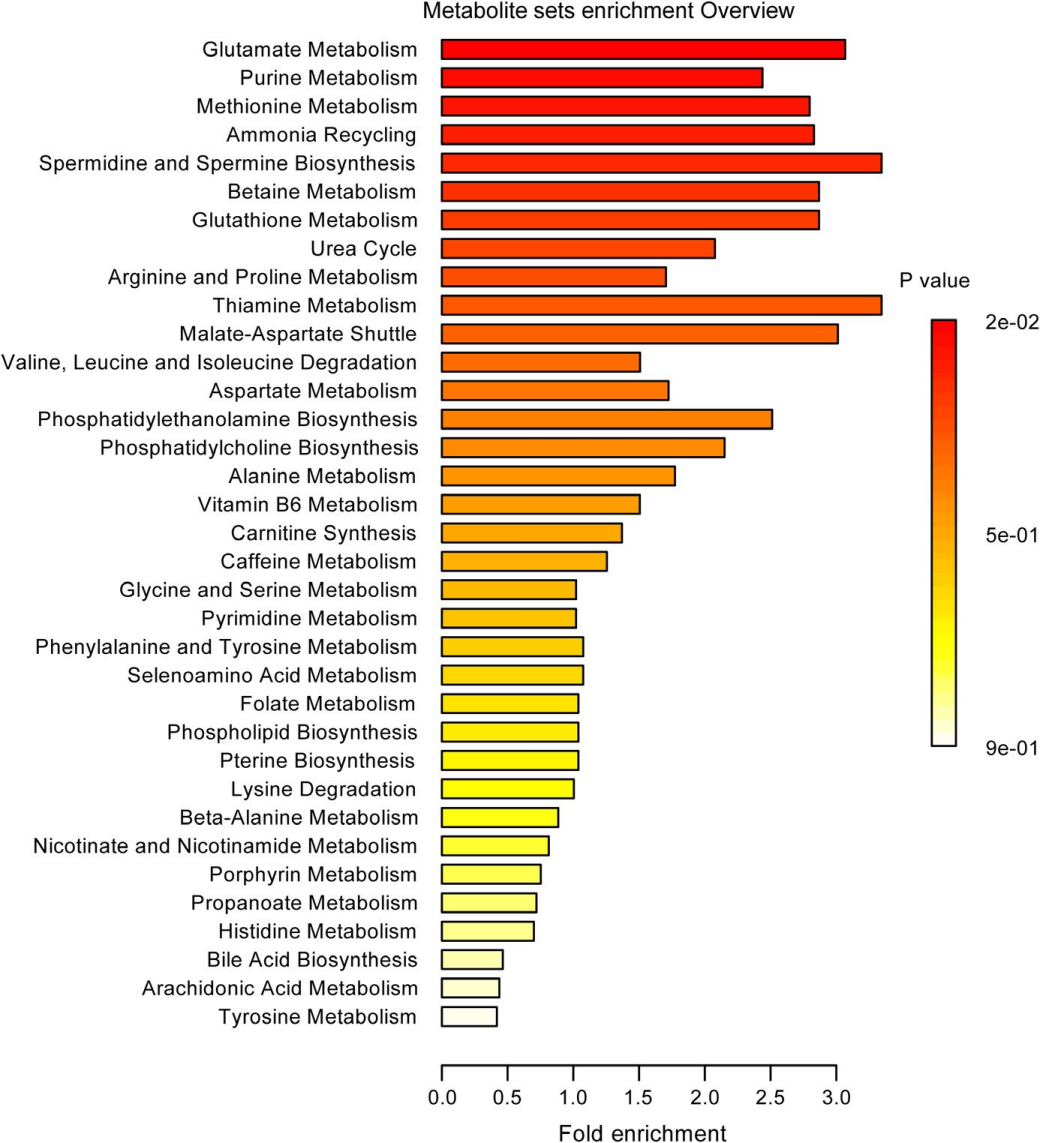
Enrichment analysis of the key metabolites in the cerebral cortex of wild‐type and PKU mice

**Figure 4:**
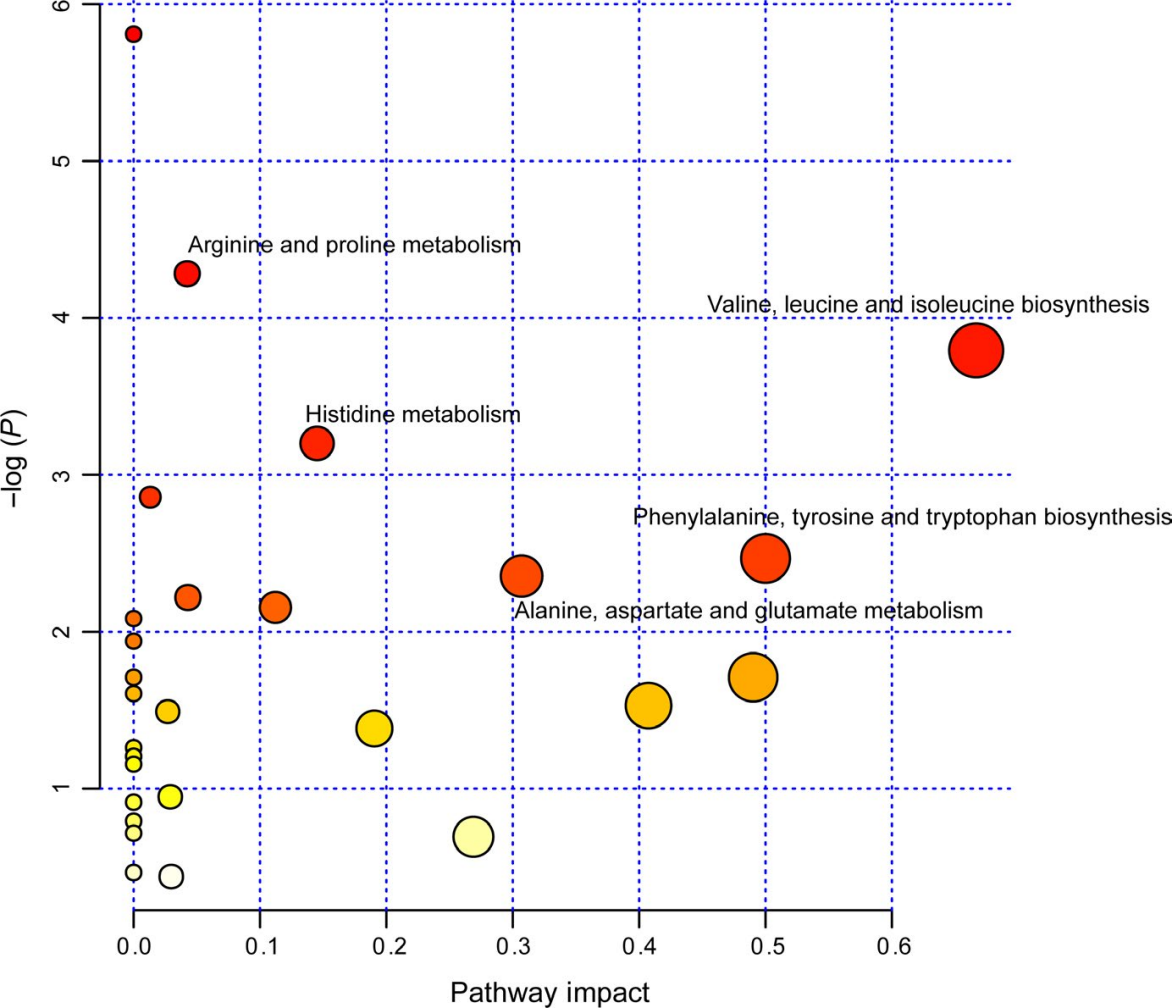
Pathway analysis of the key metabolites in the cerebral cortex of wild‐type and PKU mice. The *y* axis shows the *P*‐values and the *x* axis, the pathway impact values; the node color is based on its *P*‐value and the node size reflects the pathway impact values. Enriched metabolic pathways that had an impact > .02 were shown in the figure

Further pathway and enrichment analyses were carried out in MetaboAnalystR by placing the altered metabolites into their biochemical context. Pathway analysis referencing the KEGG pathway database revealed that a number of metabolic pathways were significantly altered in the cortical tissues of the PKU mice, mainly including phenylalanine, tyrosine, and tryptophan biosynthesis (l‐phenylalanine, N‐acetyl‐l‐phenylalanine, phenyllactic acid, 5‐methoxydimethyltryptamine, hordenine); valine, leucine, and isoleucine biosynthesis (l‐valine, l‐leucine); alanine, aspartate, and glutamate metabolism (gamma‐aminobutyric acid, l‐aspartic acid); histidine metabolism (urocanic acid, l‐aspartic acid); and arginine and proline metabolism ( 4‐guanidinobutyric acid, gamma‐aminobutyric acid) (Figure 4). Enrichment analysis referencing libraries of metabolite sets based on normal metabolism found an additional significantly altered pathway with other contributing metabolites (purine metabolism: adenine, deoxyinosine, glycine, inosine, adenosine, purine; methionine metabolism: l‐methionine, 5′‐methylthioadenosine, l‐aspartic acid) (fold enrichment ≥ 2.0, *P* < .05, Figure 3).

## 4 DISCUSSION

Phenylketonuria is an autosomal recessive disorder caused by a deficiency in the key enzyme *PAH*, which is necessary for the conversion of the amino acid phenylalanine to tyrosine. The deficiency results in excessive accumulation of phenylalanine and its derived metabolites and development of metabolic encephalopathy. However, the underlying mechanism of brain malfunction has not been completely elucidated yet.

The metabolomics method has recently emerged as a powerful tool for discovering novel diagnostic and therapeutic biomarkers of diseases, elucidating disease action mechanism and evaluating drug efficacy and toxicity by analyzing global metabolic profile changes in various tissue samples. In addition, brain metabolomics has been widely used to understand the pathology and to identify potential biomarkers for neurological diseases, such as neurodegenerative diseases, in both animal models and human postmortem tissues because metabolic changes in the brain are more likely to reflect disease etiology than metabolic changes in peripheral biofluids.^9^

In the present study, LC‐MS‐based metabolomics analysis of the cerebral cortical tissues of the *Pah^enu2−/−^* mice, an ideal mouse model for studying human PKU, revealed significant differences in many metabolites (Table S1, Table 1). Those altered metabolites are involved in many related metabolic pathways, including amino acid metabolism, purine metabolism, and methionine metabolism (Figures 3 and 4). All of these changes occurred in the developing brains of PKU mice aged 4 weeks, indicating that such metabolic disturbances might be closely related to brain dysfunction during brain development in PKU. The major metabolic patterns and plausible pathways associated with brain dysfunction in PKU mice are discussed below.

In the present study, cerebrocortical phenylalanine concentrations in PKU mice were dramatically higher than those in wild‐type control mice (Table 1), which was consistent with previous studies showing higher phenylalanine concentrations in different brain areas of PKU mice than those in the corresponding brain structures in wild‐type controls.^1^ Similar results for N‐Acetyl‐l‐phenylalanine and phenyllactic acid were also obtained in this brain region. The enrichment of these phenylalanine‐associated metabolites may be secondary to the enrichment of the precursor phenylalanine. Previous studies revealed that phenylalanine and its metabolites, such as phenyllactic acid, provoked oxidative stress in the hippocampus and cerebral cortex of developing rats and inhibited cell proliferation,^12^, ^13^ indicating that phenylalanine and its metabolite phenyllactic acid contribute to neurological dysfunction in PKU and might be one explanation for brain dysfunction in PKU.

Branched‐chain amino acids (BCAAs) are amino acids structurally characterized by the presence of aliphatic side chains. Branched‐chain amino acids in the central nervous system serve as important metabolic precursors required for the biosynthesis of proteins and neurotransmitters, and also serve as important sources of nitrogen, thereby facilitating the synthesis of such essential brain metabolites as glutamate and glutamine.^14^ The results of the present study demonstrate that the levels of valine and leucine, the two BCAAs, were significantly lower in cortical tissues of PKU mice than in those of the wild‐type controls (Table 1), suggesting that BCAA metabolism was affected in PKU. Consequently, disturbing BCAA metabolism may adversely affect brain function in PKU by interfering with the biosynthesis of essential proteins, neurotransmitters, or brain metabolites.

Another altered metabolic pathway in PKU mice was the alanine, aspartate, and glutamate pathway. The present study did not reveal an enrichment of alanine and glutamate in the cerebrocortical tissues of PKU mice (data not shown), but demonstrated that there was a significant perturbation of the metabolism pathways of alanine, aspartate, and glutamate (Figure 4) in this brain tissue. Moreover, the present study also revealed a significant enrichment of aspartate and the inhibitory transmitter GABA in the cortical tissues of PKU mice, which could be secondary to a transient enrichment of glutamate, a precursor to GABA. Additionally, a number of studies demonstrated that some amino acids, such as alanine, aspartate, GABA, and glutamate, function as neurotransmitters and/or neuromodulators.^15^ All of these findings indicate that the alanine, aspartate, and glutamate metabolic pathway could be involved in the etiology of brain dysfunction in PKU by interfering with some important neurotransmission functions.

Dysregulated purine metabolism has been repeatedly reported to be involved in the pathogenesis of several neurodegenerative diseases, such as Alzheimer's disease and Parkinson's disease.^16^, ^17^, ^18^ A recent study conducted by Alonso‐Andrés et al^18^ revealed that the alterations of purine metabolism are stage‐ and region‐dependent in Alzheimer's disease by measuring the levels of purine‐related metabolites and the activity of their converting enzymes in the frontal, parietal, and temporal cortices of patients with Alzheimer's disease at different stages of disease. Bourke^17^ reported that adenosine or guanosine loading could potentially evoke the disturbances to the A_2a_ adenosine receptors in the nigro‐striatum or to the guanosine receptors in the hippocampus, amygdala, and ventral striatum, resulting in Parkinson's disease with dementia. Theoretically, similar pathogenesis could be expected in PKU, a common inherited disease with metabolic defects and neurodegeneration. Interestingly, perturbations of purine metabolism were observed in our present study (Figure 3). Concentrations of nucleosides (adenosine, deoxyinosine, and inosine) were much higher in the cortical tissues of PKU mice than in the wild‐type control mice (Table 1), which may suggest a disturbed neuroprotective function leading to neural damage because purine nucleosides play important neuromodulator roles in the central nervous system.^19^ In addition, the abnormal recycling of brain nucleosides is finally reflected in altered levels of other purine metabolites, such as adenine (Table 1). Therefore, we speculated that, in PKU, abnormal purine metabolism may contribute to neurodevelopmental impairment.

Here, we report for the first time the differential metabolomic analysis of cerebral cortex of PKU mouse model and their wild‐type littermate controls. The exact mechanisms associated with these altered metabolic pathway in the cortical tissue of PKU mice remain unknown. Nevertheless, it should be noted that additional metabolic pathways, including arginine and proline metabolism and methionine metabolism, were found to be also dysregulated in the cortical tissues of PKU mice (Figures 3 and 4). Disruptions of these metabolic pathways were previously demonstrated to be linked with impaired neurodevelopment or brain dysfunction in various neurodegenerative or neurological diseases.^20^, ^21^ Thus, our study demonstrated that dysregulation of these metabolic pathways may to some extent contribute to the brain pathology associated with PKU.

However, it should be noted that the specific mechanisms underlying the above‐mentioned metabolic perturbations, including the enzymes and genes involved, require further investigation. This study provides a new link between PKU and metabolic pathways, including phenylalanine, tyrosine, and tryptophan biosynthesis; valine, leucine, and isoleucine biosynthesis; alanine, aspartate, and glutamate metabolism; purine metabolism; arginine and proline metabolism and methionine metabolism. These metabolic disturbances may play essential roles in the pathogenesis of brain dysfunction associated with PKU.

## 5 CONCLUSIONS

In conclusion, we report for the first time that multiple metabolic pathways were perturbed in the cerebrocortical regions of PKU mice. Some of these pathways, including phenylalanine, tyrosine, and tryptophan biosynthesis; valine, leucine, and isoleucine biosynthesis; alanine, aspartate, and glutamate metabolism; purine metabolism; arginine and proline metabolism and methionine metabolism, play essential roles in maintaining normal brain function. Therefore, the data suggest that the disturbances of these metabolic pathways might contribute to neurological or neurodevelopmental dysfunction in PKU, which could thus provide new insights into brain pathogenic mechanisms in PKU. The characterization of metabolic abnormalities in this PKU mouse model might provide novel insights into the pathological mechanisms associated with human PKU and might provide useful information for the development of the novel treatment strategy of PKU.

## CONFLICT OF INTEREST

The authors declare no conflict of interest.

6

**Table 2:** Results from metabolic pathway analysis with MetaboAnalystR

Pathway name	Total cmpd^a^	Hits^b^	Raw *P*‐value^c^	Impact^d^
Aminoacyl‐tRNA biosynthesis	69	6	.0030	0.0000
Arginine and proline metabolism	44	4	.0138	0.0424
Valine, leucine and isoleucine biosynthesis	11	2	.0225	0.6667
Histidine metabolism	15	2	.0408	0.1452
Purine metabolism	68	4	.0574	0.0131
Phenylalanine, tyrosine and tryptophan biosynthesis	4	1	.0848	0.5000
Alanine, aspartate and glutamate metabolism	24	2	.0949	0.3070
Glutathione metabolism	26	2	.1088	0.0429
Cysteine and methionine metabolism	27	2	.1160	0.1122
Cyanoamino acid metabolism	6	1	.1245	0.0000
Thiamine metabolism	7	1	.1437	0.0000
Vitamin B6 metabolism	9	1	.1810	0.4902
One carbon pool by folate	9	1	.1810	0.0000
Methane metabolism	9	1	.1810	0.0000
Nitrogen metabolism	9	1	.1810	0.0000
Valine, leucine and isoleucine degradation	38	2	.2008	0.0000
Phenylalanine metabolism	11	1	.2167	0.4074
Pyrimidine metabolism	41	2	.2253	0.0270
Nicotinate and nicotinamide metabolism	13	1	.2508	0.1905
Pantothenate and CoA biosynthesis	15	1	.2836	0.0000
Folate biosynthesis	16	1	.2994	0.0000
beta‐Alanine metabolism	17	1	.3149	0.0000
Butanoate metabolism	22	1	.3876	0.0290
Lysine degradation	23	1	.4012	0.0000
Porphyrin and chlorophyll metabolism	27	1	.4528	0.0000
Glycerophospholipid metabolism	30	1	.4886	0.0000
Glycine, serine and threonine metabolism	31	1	.5000	0.2688
Tyrosine metabolism	44	1	.6279	0.0000
Primary bile acid biosynthesis	46	1	.6445	0.0298

a Total cmpd, the total number of compounds (cmpd) in the pathway.

b Hits, the number of compounds that match with our experimental data.

c Raw *P*‐value, the original *P*‐value calculated from the enrichment analysis.

d Impact, the pathway impact value calculated from pathway topology analysis.
