# Supplementary figures and images for: Metabolomics analysis reveals perturbations of cerebrocortical metabolic pathways in the Pahenu2 mouse model of phenylketonuria
Source: CNS Neurosci Ther. 2019 Aug 31;26(4):486–93. doi: 10.1111/cns.13214 (PMC7080435; doi:10.1111/cns.13214)

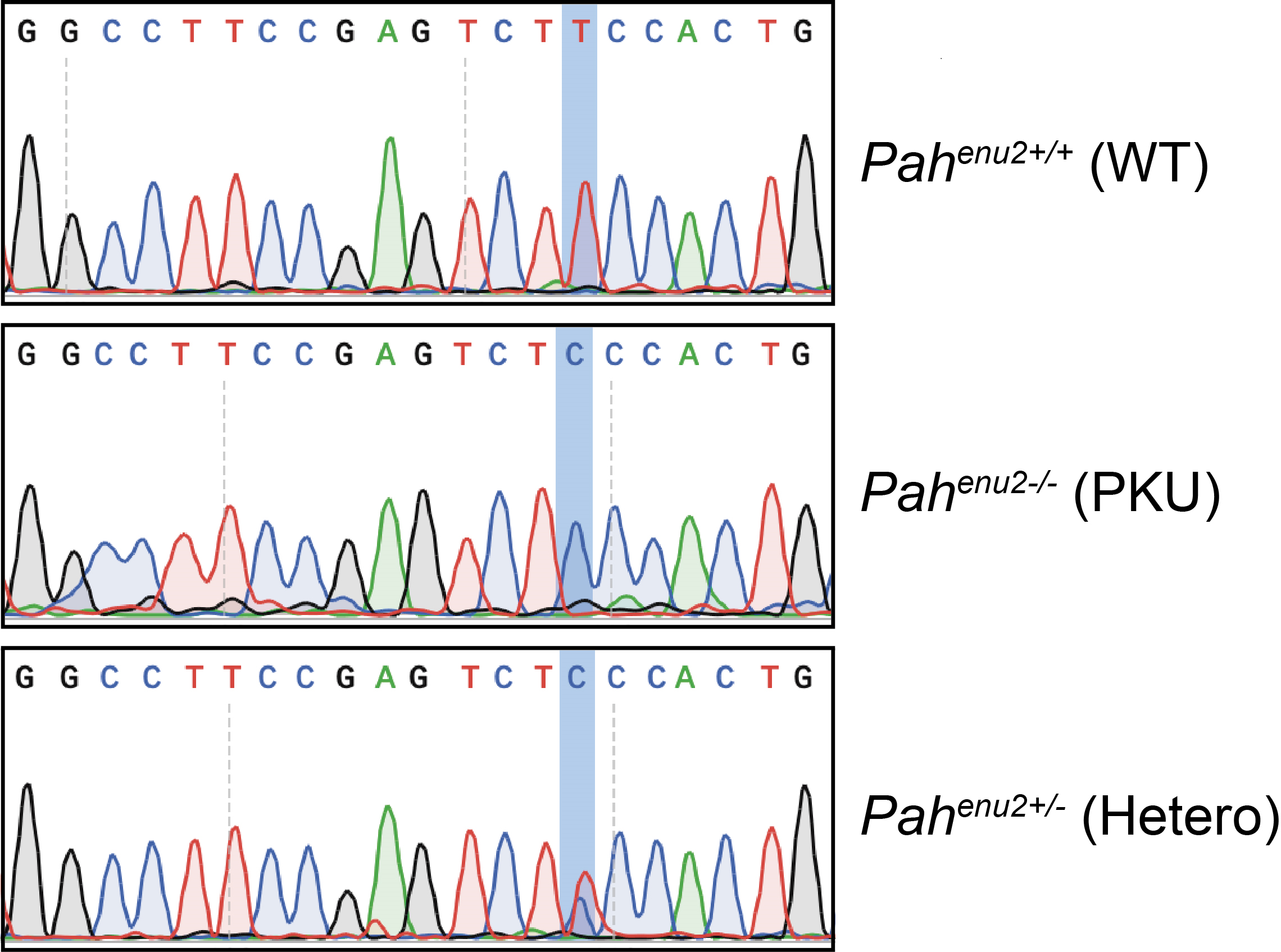

Supplement: Supplementary file 1 [file CNS-26-486-s001.tif]
